# "The Hospital" Medical Book Supplement.— No. VIII

**Published:** 1908-06-20

**Authors:** 


### "THE HOSPITAL"
### MEDICAL BOOK SUPPLEMENT.—No. VIII.

CONTENTS.

**Medicine—**
Diseases of the Nervous System ... 317
The Medical Annual, 1908 ... 317

**Tropical Medicine—**
Fevers in the Tropics: their Clinical and Microscopical Differentiation, including the Milroy Lectures on Kala Azar ... 318
A Guide to Sick Nursing in the Tropics ... 318

**Gynæcology and Obstetrics—**
A Short Practice of Midwifery... ... 318

**Therapeutics—**
Diets in Tuberculosis ... 319
Electrical Treatment ... 319
Air, Light, and Sun Baths in Treatment of Chronic Complaints 319

**Pediatrics—**
Infancy and Infant Rearing ... 320

**Miscellaneous Items—**
Rummage ... 320
A Pocket Codex ... 320
A Book of Poems ... 320
A Useful Pocket Formulary ... 320

### MEDICINE.

DISEASES OF THE NERVOUS SYSTEM. By H. CAMPBELL THOMSON. Pp. 480. (Cassell and Company. Price 10s. 6d.)

THERE is a distinct opening for a book on nervous diseases suitable for the junior student. Larger text-books too often oppress the beginner with their complexity. Dr. Campbell Thomson has written a capital compendium of nervous diseases which will be found useful by the class of readers for which it is designed. The book is divided into five sections. The first section is introductory, and commences with a short account of the general structure of the nervous system. Then follow chapters on reflexes, rigidity and contractures, electrical reactions, and lumbar puncture. Though somewhat handicapped by the condensed style in which they are treated, they give the student, on the whole, a very fair conception of these subjects. The writer states his views dogmatically, and rightly so, for though one may sometimes disagree with his statements, we recognise that to the beginner dogmatism is an advantage; it gives him a working basis on which to build his knowledge. For example, on the opening page axons are defined as processes which conduct impulses away from the cell body. A notable exception to this rule occurs, of course, in the case of the peripheral sensory nerves, whose fibres conduct towards the cells of the posterior root ganglia.

The second section deals with affections of the peripheral nerves. The cervical sympathetic is grouped with paralyses of the upper limb—an arrangement which may be convenient clinically, but is somewhat illogical, since the cervical sympathetic may be affected at many other parts of its course than where it accompanies the roots of the brachial plexus. No allusion is made to the abdominal sympathetic or its diseases.

The third section is devoted to the myopathies and to the peroneal type of atrophy. The fourth section, on diseases of the spinal cord, is concisely written, and presents a good summary of current views on these maladies. Here again the author is somewhat hampered by the condensation of his subject. The account of intermittent claudication, for example, is inadequate, gangrene of the feet being a very much rarer complication than the reader would be led to suppose from Dr. Campbell Thomson's description. Moreover, most authorities would be inclined to class this disease with the vaso-motor affections. The different diseases of the spinal cord are clearly and concisely described.

The fifth section, on organic diseases of the brain, includes the various forms of meningitis, the cerebral palsies of children, cerebral abscess, intra-cranial tumours, cerebral vascular lesions, and general paralysis of the insane. No mention is made of the diagnostic value of examination of the cerebro-spinal fluid in doubtful cases of general paralysis. Aphasia is well described, and an account is given both of Marie's views and of the hitherto orthodox doctrines on this subject. Dr. Campbell Thomson, however, does not state to which of the sides he himself leans.

The sixth and last section discusses diseases of functional and general origin, such as myasthenia, tetany, epilepsy, paralysis agitans, chorea, tics, the various neuroses, etc. Vertigo is described in a special chapter. The final chapters are on vaso-motor neuroses, exophthalmic goitre, tetanus, and hydrophobia.

The subject-matter is good, and the book will form a useful introduction to the study of nervous diseases.

---

THE MEDICAL ANNUAL, 1908. (J. Wright and Company, Bristol. Pp. 714. Price 8s. 6d. net.)

THE "Medical Annual" occupies an established position in professional literature. The recently issued edition maintains throughout those features on which have been based the popularity of previous issues. Not only are summaries of the year's researches drawn up by experts in their various departments, but fairly successful attempts are made to notice at length important papers and to deal more briefly with ephemeral productions and with immature and irresponsible opinions. Moreover, there are also original articles on various matters on which the progress of scientific knowledge invites authoritative pronouncements. In addition, short paragraphs embodying practical suggestions for treatment from previous Annuals are given in connection with various diseases which do not happen to have come under discussion during the year. These notes are printed in smaller type, and are confined rigidly to brief and pointed synopses; hence they do not materially increase the bulk of the work, while they add distinctly to its usefulness. Perhaps one of the most abiding impressions obtained by perusing the pages of this book is that of the large number of much-boomed remedies and treatments which have been proved by the experience of a year or two to be futile. Probably back numbers of the Annual would be instructive reading in this respect; there one would doubtless find the sanguine claims of the inventors of various discarded methods set out among the newest work of the day. How many of those here recorded will emerge triumphantly from the test of time, no editor, of course, can say. It is something to be thankful for that we have set down in clear and intelligible form year by year in the "Medical Annual" the claims of their discoverers and the subsequent investigations of less prejudiced people.

### TROPICAL MEDICINE.

Fevers in the Tropics: Their Clinical and Microscopical Differentiation, Including the Milroy Lectures on Kala-Azar. By Leonard Rogers, M.D., F.R.C.P., F.R.C.S., B.S., I.M.S., Professor of Pathology, University of Calcutta. (London: Henry Frowde and Hodder and Stoughton. Pp. 343. Price 30s. net.)

In this book is contained an exceedingly interesting and complete account of many of the fevers which are specially prone to occur in India and other parts of the tropics. They are all discussed according to the most recent scientific tests, one of the chief features being the work done on the different blood changes found in them. To tropical experts, and no less to the profession at home, there are many points of interest spread through the book. Why scarlet fever and diphtheria should find a difficulty in maintaining their position in hot climates offers an interesting field for study. Relapsing fever, a disease sometimes met with in Europe, is common, and Vandyke Carter wrote on it as long ago as 1882. Since that date another form, known by the name of African tick fever, has been described in the adjoining continent, and a short account of it is appended. Here we notice that the proof-reader has not been so attentive to the spelling of proper names as he might have been. Some people suffer annoyance by having their names mutilated, so this point might be attended to in later editions. The Milroy lectures on Kala-Azar recently delivered in London appear under the chapter bearing that name. A new point in treatment brought out is that ipecacuanha has a curative effect on latent amœbic infections of the liver and bowels; the application of this drug to cases of hepatitis before an abscess has actually formed may prevent its occurrence. This is very important for practitioners at home as well as abroad, for many cases of this disease are constantly returning home from India. Under the title "Seven day fever" a new clinical entity is described. Bearing some resemblance to dengue, it would certainly appear from the account given of it to be different, and, further, the author has in some instances isolated a germ of the coli-typhoid group from the blood. It is not influenza, which is common enough in Calcutta, so we may add it to the list of new diseases. Unfortunately the changes in the blood vary considerably, so they are of little help in the diagnosis. More information concerning this new disease will be eagerly awaited, and much credit is due to Professor Rogers for having described it, and differentiated it from malaria and other indigenous fevers. Malaria has, of course, a chapter devoted to it, and a very good description of the disease as seen in India is given. It is a pity more space could not have been devoted to blackwater fever, as this is of special interest and importance, more, of course, perhaps in Africa than in India. We note also that, contrary to general opinion, the author believes that quinine by the mouth is as quickly absorbed as by intra-muscular injection. The only blemish about the book as a whole is that all the work is Indian, and the title, "Fever in the tropics," is in some ways a misnomer. Still, allowing for this, there is undoubtedly a vast amount of valuable material contained in it, which should prove of great use both to Indian practitioners and others practising in different parts of the tropics.

---

A Guide to Sick Nursing in the Tropics. By Andrew Duncan, M.D., F.R.C.P. (London: The Scientific Press. 1908. Pp. 162. Price 2s. 6d. net.)

In tropical climates the trained nurse is especially valuable in the treatment of the sick. The actual nursing of the patient presents many problems which have rarely to be faced in this country. Heat, flies, sandstorms, mosquitoes, and all the other discomforts of life in the tropics aggravate tenfold his condition and the difficulty of treating him, especially as in very many places the resources of civilisation are unprocurable. But even more important than the instruction of the nurse in relieving her patients and attending to their comfort by forethought and skill is her expert assistance in successful prophylaxis. So many diseases rife in the tropics, especially in India and other densely populated parts of the East, occur in epidemics, that the first thought of the medical man must always be how to prevent his case from infecting others. The obstacles are often immense, owing to the ignorance or religious prejudice of natives, and, in fact, may be insuperable; but the physician fortunate enough to be assisted by nurses who have thoroughly absorbed Dr. Duncan's teaching on these vitally important questions will be in a splendid position to carry out the necessary measures. There are also valuable hints on the preservation of the nurse's own health, and these no nurse, nor anyone else, should neglect. Dr. Duncan has his own views on many of the still disputed points in connection with various tropical diseases, but he does not omit to explain also the ideas of those who hold differently. He has made his pages readable and interesting not only to nurses and medical men, but to all those whose lot is cast in the tropics, and the book can be recommended. We could wish the proofs had been more carefully read; the frequent lapses from our own tongue are most exasperating.

---

### GYNÆCOLOGY AND OBSTETRICS.

A Short Practice of Midwifery. By Henry Jellett, M.D., F.R.C.P.I. 5th Edition. (London: J. and A. Churchill, 1908. Pp. 654, with 200 illustrations. Price 10s. 6d. net.)

The appearance of the fifth edition of the shorter of Dr. Jellett's two text-books of midwifery is evidence of the favour with which it has been received since the first edition 11 years ago. The present issue has been extensively revised, and the general arrangement brought into line with that of the larger work. The plan of designing separate and distinct works to satisfy the needs of students and practitioners respectively is one which might be more widely adopted, and is infinitely preferable to the endeavour to serve both classes in a single volume, as is so often attempted. To the student pathology, classification, and examination lore in general are the more important; to the practising medical man treatment and practical details of every sort are of greater value than the ætiology of placenta prævia or the diagrammatic representation of the fœtal circulation, let us say. Dr. Jellett in the main fulfils here the requirements of the student very well indeed. He is not over discursive nor too full of references; he is clear and intelligible, speaks with authority, and he has chosen his diagrams with discretion. His "Short Practice" may be recommended with confidence to those about to submit to the examination test of their acquaintance with the science of midwifery. One feels a doubt whether there is much to be gained by recommending to students the palpation of the enlarged ureters as a sign of pregnancy, but in other respects the author has omitted the recondite and retained the essential excellently.

It is not surprising that criticism should here and there arise; partly because there are a few traditional variations of practice between the two sides of St. George's Channel, partly because a small text-book of an important science cannot please everyone. Thus we should prefer to see some reference to the trinitrin treatment of eclampsia, and we think that the average duration of the second stage of normal labour is somewhat longer than Dr. Jellett's experience leads him to believe—that is, one to two hours in primiparæ and ten to fifteen minutes in others. The repair of torn perinæums is less lucidly dealt with than might be expected from Dr. Jellett's admirable descriptions of operations in his work on gynæcology. It is noteworthy that the rational system of dating the events and course of pregnancy by lunar months is throughout adopted, and, in view of differences of opinion abroad, that the uterus is described as reaching the umbilicus at the end of the sixth (lunar) month. The agreement of the author with British teaching as opposed to that of certain foreign authorities on other questions is also evident. Thus Dr. Jellett is not in favour of leaving the third stage of labour entirely to nature, as is advocated by von Winckel, for example, who says that 86 per cent. of placentæ are spontaneously delivered within an hour. He also teaches the engagement of the vertex three or four weeks before labour in primiparæ, a proposition which has been recently contested in America; and the question of ectopic gestation is another one on which Irish views seem to coincide with those held in this country. It is of interest to notice that the author condemns both the high-forceps operation and the oscillation of the forceps blades often advised in British text-books; the latter is considered to be potential of serious injury to the maternal passages. Taken all round, there are few text-books on this subject of equal size which convey so complete and satisfactory an outline of midwifery as does this.

### THERAPEUTICS.

Diets in Tuberculosis. By Noel D. Bardswell and J. E. Chapman. (Frowde, Hodder and Stoughton. London. 1908. Cr. 8vo. Pp. 184. 6s. net.)

Messrs. Bardswell and Chapman are to be heartily congratulated on this excellent book. It is concisely written, and is practical from cover to cover. The task which the authors set themselves was to determine in the first place the nutritive value of the diet best suited for the treatment of the average consumptive, and secondly, the best lines upon which such a dietary can be economically constructed for actual use. In other words, they have set to work to discover empirically the cheapest form in which a suitable diet can be presented to consumptive patients. The importance of this research needs not to be laboured. As the authors point out, a saving of 5d. per head per day on food means, in the case of a sanatorium of fifty beds, an economy of £350 a year; and every hospital physician will recall many instances in which he has felt the futility of advising some impoverished creature to take two quarts of milk a day, with plenty of butter and other nourishing food. The laborious researches of the writers, based upon clinical experience, show that by care in selection of materials and in the cooking of them, a diet suitable for consumptives both in nutritive value and in palatability can be provided at a cost varying from 8d. to 11d. a day. Economy is effected by replacing whole milk by separated milk, which is of no mean nutritive value, by using the cheapest cuts of meat, by increasing the proportion of bread and pulses, and by substituting margarine for butter. The book is richly supplied with full menus and practical instructions for cooking, and deserves the highest praise.

---

Electrical Treatment. By Wilfred Harris, M.D., F.R.C.P. Pp. 383. (Cassell and Co. Price 7s. 6d.)

This excellent handbook is more than a mere treatise on electro-therapeutics; it teaches the practitioner not only how and when to employ the various forms of electricity, but, what is of equal importance, it also tells him when not to use them. Moreover, apart from electrical treatment, it is full of useful hints in the management of nervous maladies. The book is essentially practical and particular attention is given to results attainable by the practitioner by means of a good faradic and galvanic battery, without necessarily investing in more complicated and expensive apparatus. An introductory chapter on methods and apparatus is followed by a clear account of the faradic current and of the various morbid conditions for which it may be beneficially employed. The utility of these chapters would be materially enhanced had diagrams been provided of the common position of the chief "motor points." Doubtless this will be remedied in a future edition. Then follows a description of the galvanic current and of the diseases in which galvanism is of therapeutic value. Amongst the illustrative cases it is curious to find the author, under the heading of "traumatic neurasthenia," giving particulars of two cases (pp. 58 and 59) which are examples not of neurasthenia but of hysteria. A useful chapter treats of electrolysis and the electrical treatment of nævi, "superfluous hairs and moles" (are not all moles superfluous?) uterine fibroids, aneurysms, etc. The last four chapters deal with the more elaborate electro-therapeutical appliances, necessitating a more complex instrumental installation than the ordinary practitioner is likely to possess. Thus there are good descriptions of the various forms of electric baths, the sinusoidal current and its medical applications, electric-light baths, and the use of *x*-rays. The only omission of importance in this latter category is the treatment of syringomyelia by means of *x*-rays, a method which has recently proved of benefit in a number of cases. In some of his anglicised versions of Greek derivatives the writer is not strictly logical; thus, for example, he speaks of "cataphoresis" on the one hand, and of "kat-electrotonus" on the other. These flaws, however, are but minor affairs, and they do not detract from the value of this highly practical work.

---

Air, Light, and Sun Baths in the Treatment of Chronic Complaints. By Dr. A. Montennis. Translated from the French by Fred Rothwell. (London: John Bale, Sons, and Danielsson, Ltd. 1907. Pp. 73. Not illustrated. Cap. 8vo. Price 2s. net.)

This brochure is not, as its title might lead one to think, a brief for any particular establishment in which the treatment referred to is carried on. It is a *bona fide* appeal to the public to take greater care of themselves in respect to rational exercise, and to adequate participation in God's gifts—sunlight and fresh air. It is pointed out that the treatment by open air and sunlight by no means necessarily requires resort to a foreign spa; the treatment can be carried on daily and regularly at home if only a little trouble about it be taken. For the most part the advice given is sound common sense, with particular attention to small details; here and there, however, there is a tendency to exaggerate procedures, such as going out of doors barefoot for example. Medical men will read the book with interest, and they will be glad that their patients should do so likewise, because their doing so will help the doctor to get his advice carried out. The gist of the book is given by the quotation from Michelet: "Of all flowers the human flower is the one that needs sunshine most."

### PAEDIATRICS.

Infancy and Infant Rearing. By J. B. Hellier, M.D. Second edition, revised. Pp. 164; 29 charts and illustrations. (Charles Griffen and Co. Price 3s. 6d.)

The fact that this introductory manual has reached a second edition is a definite indication of value. It supplies with a reasonable amount of information, expressed in non-technical language, the class of persons for whom it is avowedly written, namely pupil midwives, nurses, those engaged in teaching the care of infants, medical students and practitioners. In catering for such a huge class the requirements of each group are unequally filled; some get too rich a mental pabulum, while for others the food is a little too simple. Still, even the practitioner will obtain valuable common-sense information on subjects which are neglected in the ordinary medical curriculum. The details of growth and development, the various methods of feeding, the hygiene of infancy, the care of premature infants, the rearing of infants in tropical climates, and the prevention of infantile mortality are dealt with in a simple, sensible, and rational manner. As appendices there are included schedules of diet for different periods of infancy, the work of health visitors, and the signs of disease in the first ten days of life. On the whole, the author has carried out his scheme successfully, and he has not fallen into the fatal temptation of entering into details of medical treatment. We can strongly recommend the book to all who are interested in health visiting and the reduction of infantile mortality. Perhaps for this class the writer could make his work even more valuable by the omission of details and references suitable for the profession, and by rather greater simplicity in the directions for artificial feeding.

### MISCELLANEOUS ITEMS.

**Poetry and Medicine.**

"Songs of the Downs and Dunes," by Habberton Lulham, is the title of a book published by Kegan Paul, Trench, Trübner and Co., at the price of 3s. 6d. Compared with the other learned professions, medicine contributes comparatively few of its members to the service of contemporary literature. Notable exceptions there are, it is true: for instance, Mr. W. S. Maugham, four of whose plays are now running in London simultaneously, is a qualified medical man, and so is the anonymous writer of "Confessio Medici." But in the main we do not as a class shine in the craft of authorship. Whether this is due to anything inherent in the study of medicine is not easy to decide. It may be argued that the scientific training, the subordination of everything to sober truth and orderly method, checks that development of the fancy and of the imagination which marks the creative artist. Or it may be that medicine is a mistress so entrancing that no other may rival her charms, and certainly that is so with some of her worshippers; or that her service is so exacting that there is no time nor opportunity for dalliance. So much the more do we congratulate ourselves, therefore, on the achievement of anyone who does step out of the ranks with a creditable piece of literary work. For Mr. Lulham's verse is always marked by distinction and loftiness of thought, and not seldom rises to a very high level of poetic insight and lyrical excellence; moreover, it is original both in thought and expression and technically almost flawless. There is a dominant chord of sorrow through many of the shorter songs which imparts a certain dignity to their melody; but the author can handle themes gay as well as grave, and his love-songs are many of them very good indeed. In the longer blank verse pieces the rhythm halts a trifle awkwardly on occasions, which is the more surprising since, for the most part, elsewhere Mr. Lulham pleases the ear as much as he does the understanding. On p. 8, for example, there is an exquisite song which well deserves a setting from some master of music; and the first sonnet, entitled "Reveillé," is a fine effort of noble and sustained imagery, and of sonorous and musical diction. Indeed, but for the one blot of a shocking rhyme, it is probable that this lyric might take rank among the very best sonnets in the language. For the rest there is a tiny poem to a bootblack which pleases us greatly and conveys a subtle hint of Calverley in more than the title; but there are also many others that are well worth the attention of anyone who cares about either the downs and dunes or contemporary English poetry.

**A Book of Essays.**

A small book entitled "Rummage," by H. C. P. Pattin, is published by Sidney Appleton at the price of 2s. net. This is a collection of ten little essays by a medical man with a taste for literary expression. Two or three of them deal more or less directly with professional matters; the rest are slight random sketches and impressions of merely general interest, and with no particular relation to each other. The author writes in a personal and reminiscent manner. His style is light, and would be graceful were it not marred by occasional wayward turns of expression. The character study of Sir George Humphry is admirable, and by itself would justify us in recommending Dr. Pattin's little volume as a companion for an idle hour.

---

**A Useful Pocket Formulary.**

The little "Pharmacopœia for Diseases of the Skin," edited by Mr. James Startin and published by John Wright and Co., of Bristol, at the modest price of half-a-crown, is an exceedingly useful aid, both for practitioners and students. The sixth edition which has just been issued has been revised in a most thorough manner, and some excellent prescriptions have been added. The arrangement is almost ideal, and to each formula are attached a few simple and easily understood directions, together with a note of the skin affections in which it may prove of service. At the end is a classified list of skin diseases with notes on treatment. Such a work, though it can never replace the experience actually gained in "Skin Out-patients," has nevertheless a large field of usefulness, and will win hearty approval from those who peruse it.

---

**A Pocket Codex.**

We have received from Messrs. Evans, Gadd and Co., Ltd., wholesale and export druggists, of 97-100 Fore Street, Exeter, a copy of their "Pocket Book and Diary for Physicians and Pharmacists," edited by Mr. H. Whippell Gadd. The chief feature of this little book is a summary of the principal preparations contained in the recently published B.P. Codex. The summary is excellently made, and each drug is easily found. Future editions of what seems to be a useful little manual should be bound in limp cloth or leather: the binding of the present edition is far too stiff and angular for convenient pocket use.

